# Analysis of tumor microenvironment composition in vestibular schwannomas: insights into NF2-associated and sporadic variations and their clinical correlations

**DOI:** 10.3389/fonc.2024.1340184

**Published:** 2024-05-16

**Authors:** Vera Nickl, David Ziebolz, Charlotte Rumpel, Dennis Klein, Robert Nickl, Eva Rampeltshammer, Camelia M. Monoranu, Ralf-Ingo Ernestus, Cordula Matthies, Mario Löhr, Carsten Hagemann, Maria Breun

**Affiliations:** ^1^Department of Neurosurgery, Section Experimental Neurosurgery, University Hospital Würzburg, Würzburg, Germany; ^2^Department of Neurology, Developmental Neurobiology, University Hospital Würzburg, Würzburg, Germany; ^3^Institute of Pathology, Department of Neuropathology, University Würzburg, Würzburg, Germany

**Keywords:** vestibular schwannoma, tumor microenvironment, repair cells, immune cells, neurofibromatosis type 2

## Abstract

**Objective:**

Vestibular schwannomas (VS), benign tumors stemming from the eighth cranial nerve’s Schwann cells, are associated with Merlin gene mutations, inflammation, and the tumor microenvironment (TME), influencing tumor initiation, maintenance, and potential neural dysfunction. Understanding TME composition holds promise for systemic therapeutic interventions, particularly for NF2-related schwannomatosis.

**Methodology:**

A retrospective analysis of paraffin-embedded tissue from 40 patients (2013-2020), evenly divided by neurofibromatosis type 2 status, with further stratification based on magnetic resonance imaging (MRI) progression and hearing function. Immunohistochemistry assessed TME components, including T-cell markers (CD4, CD8, CD25), NK cells (CD7), and macrophages (CD14, CD68, CD163, CCR2). Fiji software facilitated image analysis.

**Results:**

T-cell markers (CD4, CD8, CD7) exhibited low expression in VS, with no significant NF2-associated vs. sporadic distinctions. Macrophage-related markers (CD14, CD68, CD163, CCR2) showed significantly higher expression (CD14: p = 0.0187, CD68: p < 0.0001, CD163: p = 0.0006, CCR2: p < 0.0001). CCR2 and CD163 significantly differed between NF2-associated and sporadic VS. iNOS, an M1-macrophage marker, was downregulated. CD25, a regulatory T-cell marker, correlated significantly with tumor growth dynamics (p = 0.016).

**Discussion:**

Immune cells, notably monocytes and macrophages, crucially contribute to VS pathogenesis in both NF2-associated and sporadic cases. Significant differences in CCR2 and CD163 expression suggest distinct immune responses. Regulatory T-cells may serve as growth dynamic markers. These findings highlight immune cells as potential biomarkers and therapeutic targets for managing VS.

## 1 Introduction

Vestibular schwannomas (VS) are benign tumors of the eighth cranial nerve caused by Schwann cell proliferation (1). A major characteristic in VS pathogenesis is the loss of merlin protein function. Merlin is a transmembrane protein that links cell membrane and cytoskeleton. It is activated by intercellular adhesion and by attachment to the extracellular matrix (2). In patients with NF2-related schwannomatosis (NF2), one allele for merlin on chromosome 22 is defective at birth, and a second hit during lifetime may result in loss of protein function. As hallmark tumors these patients develop bilateral VS at a young age. Later on, several other tumors, such as meningioma and ependymoma develop in addition in most cases (3, 4). Surgery, radiosurgery and “watch & wait” are the currently established therapeutic options for VS. However, in patients with NF2, these treatment options are very limited due to the higher invasiveness of the tumors and cumulative morbidity after multiple interventions and because of tumor burden. Bevacizumab might be an additional therapeutic choice. However, for this subgroup of VS as well as for older patients with high comorbidity, a better systemic therapeutic approach is urgently needed (3).

To understand genesis, sustenance and growth of VS, the surroundings of the tumor have to be taken into account. Inflammation and tumor microenvironment (TME) might play a crucial role in tumor formation and maintenance, in nerve infiltration, dysfunction and neuropathy. Understanding the composition of the TME will also benefit in the research of a potential systemic therapeutic approach not only for patients suffering from VS, who are not suitable for surgery due to age, comorbidity, or size of the tumor, but even more importantly for patients suffering from NF2. Identifying different cellular components of the TME, their quantity and localization might give us a better idea of the VS-TME interaction. The immune cell population consists of tumor-associated macrophages (TAMs) as well as CD4+ and CD8+ T-lymphocytes, regulatory T-cells (CD25+) and Natural Killer (NK) cells (CD7+). TAMs are derived from circulating monocytes and can be polarized into different functional phenotypes, primarily classified as M1 and M2. CCR2 identifies macrophages that immigrated to the central nervous system. Immune cell infiltration tends to happen in rather loosely cellular Antoni B areas (5–7). TAMs play an important role in solid tumors (8, 9) and drive regulating processes like growth and tumor invasion (8, 9). Depending on surrounding cytokine expression, macrophages show a broad variety of functional stages. Here, we focus on the investigation of the pro-inflammatory M1-macrophages and the immune-regulating M2-macrophages (10). While M1-macrophages show anti-tumor effects and are associated with a better prognosis in many solid tumors (11), M2-macrophages promote tumor growth and angiogenesis (12). Macrophage expression in VS had a significant impact on tumor growth (13) and tumor vessel density (14). Information on the role of lymphocytes in the VS TME is scarce. An investigation of 10 NF2-associated VS showed perivascular expression of lymphocytes (15). However, their role remains unclear.

Nuclear c-jun is a marker for Büngner cells, a Schwann cell in repair mode, which are an additional part of the TME beside immune cells (16). It is expressed in immature Schwann cells but downregulated in mature myelinating cells. Following injury, c-jun is upregulated and phosphorylated in Schwann cells, driving a demyelination program. Recent studies have highlighted the significance of c-jun in nerve injury response, as its conditional ablation in Schwann cells leads to increased neuronal death, impaired axonal regeneration, and diminished functional recovery (17). Molecularly, c-jun regulates the expression of myelin structural genes such as MBP and P0, as well as trophic factors like BDNF and GDNF, essential for regeneration (17). Additionally, c-jun activation induces expression of genes distinct from those in immature Schwann cells, indicating a specialized role in injury response. Paracrine signaling mediated by c-jun-induced GDNF and Artemin is crucial for neuronal survival post-injury (17). Importantly, c-jun’s involvement appears specific to the injury response, suggesting its essential role in peripheral nerve regeneration. Moreover, aberrant c-jun activation in peripheral neuropathy patients underscores its potential pathological significance. C-jun has been implicated in the context of Büngner cells within VS. These specialized Schwann cells contribute to the tumor microenvironment and play a pivotal role in tumor progression. C-jun has been shown to modulate various cellular processes, including proliferation, differentiation, and survival, and its aberrant activation has been associated with tumor growth and invasion. In VS, c-jun may influence the behavior of Büngner cells by regulating their response to local signaling cues and promoting their participation in tumor-stromal interactions. Understanding the role of c-jun in Büngner cells could shed light on the mechanisms underlying tumor-stromal crosstalk and provide insights into potential therapeutic targets. Further investigation is warranted to unravel the precise molecular pathways through which j-jun influences Büngner cells and its implications for VS progression (16). The aim of this retrospective analysis was to investigate the composition of the cellular TME and c-jun expression in VS in 40 patients and to determine possible correlation with clinical data. Differences between sporadic and NF2-associated VS will be investigated. Certain types of immune cells, present specifically in VS, could serve as targets for immunotherapeutic approaches in the future.

## 2 Materials and methods

### 2.1 Tissue samples

**Figure 1:**
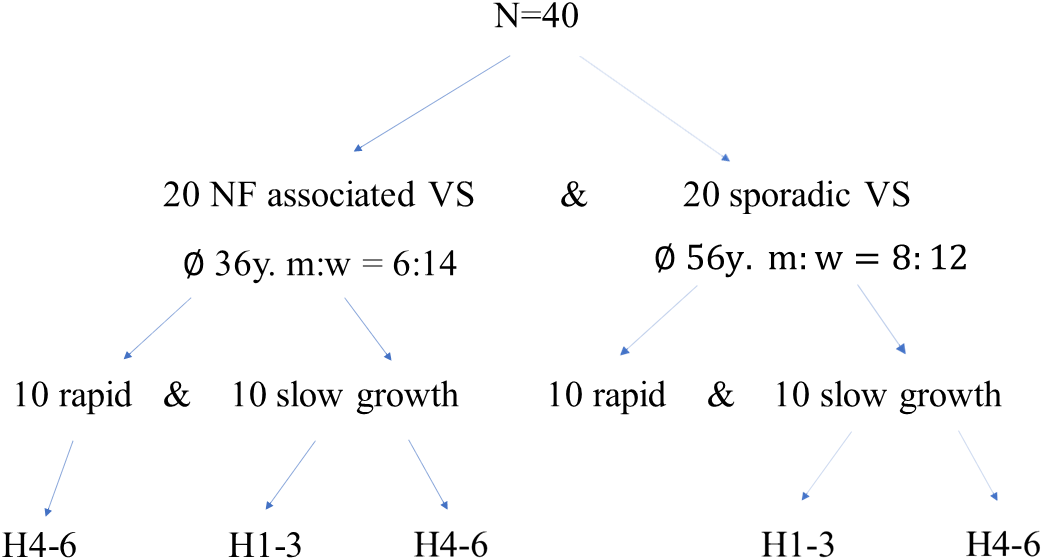
Patient cohort characteristics: Initially, a cohort comprising 40 patients was assembled, with 20 individuals presenting NF2-related schwannomatosis (NF2-associated VS) and an equal number diagnosed with sporadic vestibular schwannoma (sporadic-VS). Subsequently, these patient groups were further stratified based on tumor progression dynamics (slow vs. rapid growth) and hearing function using the Hannover classification system (19). NF2, neurofibromatosis type 2; VS, vestibular schwannoma; m, male; f, female; H1-H3, serviceable hearing; H4-6, bad to lost hearing.

All patients included in this retrospective analysis were treated between 7/2013 and 7/2020 at the Department of Neurosurgery, University Hospital Würzburg, Germany. The histopathology of the tumor samples was confirmed by an experienced neuropathologist and classified according to 2021 WHO criteria (1). Our patient selection process was conducted retrospectively and consecutively, representing a backward progression in time until we reached a cohort of 10 patients per group, categorized as sporadic VS slow-growing, sporadic VS rapid-growing, NF-2 slow-growing, and NF-2 rapid-growing (**Figure 1**). We aimed to achieve balance by ensuring an equal distribution of 10 patients in each group. Two autoptic vestibular nerves from subjects with no tumor served as control nerves. Tumor progress was determined by magnet resonance imaging (MRI) and slow progress defined as less than 2 mm growth within up to 1 year (18). Furthermore, patients were stratified for hearing impairment according to the Hannover classification (19) (**Table 1**).

### 2.2 Immunohistochemical staining

For immunohistochemistry, tissue was cut to a thickness of 2 µm, deparaffinized in xylene and hydrated in a graded series of alcohols. Heat induced retrieval was performed for eight (CD4, CD8 CD68, CD163) or ten (CD14, CCR2) minutes with citrate buffer (pH = 6.0). Alternatively, heat induced retrieval was performed in Tris-EDTA buffer (pH = 6.1) for three (CD7, CD25) or Tris-EDTA buffer (pH = 9.0) for five minutes (iNOS). Slides were blocked with 30% hydrogen peroxide (PanReac Applichem, ITW Reagents). Thereafter, the primary antibody was applied over night at 4°C (CCR2) or one hour at room temperature (CD4, CD7, CD8, CD14, CD25, CD68, CD163 and iNOS): CD4 (M7310, Dako, clone: 4B12, dilution: 1:200), CD7 (M7255, Dako, clone: CBC37, dilution: 1:500), CD8 (M7103, Dako, clone: C8/144B, dilution: 1:80), CD14 (ab183322, abcam, clone: SP192, solution; 1:800), CD25 (MA5-12680, Thermo Fisher, clone: IL2R.1, dilution: 1:40), CD68 (AG Brentlein, clone: KiM1P, dilution: 1:1000), CD163 (NCL-CD163, Leica, clone: 10D6, dilution: 1:800), CCR2 (ab176390, abcam, clone: 7A7, dilution: 1:400) iNOS (ab239990, abcam, clone: SP126, dilution: 1:50). Antibodies were diluted with Dako FLEX ANTIBODY DILUENT (DM830) and for CCR2 with Zytomed Antibody Diluent (ZUC025-500).

Slides were then incubated with the secondary antibody for 20 minutes and labeled (HiDef Detection™ HRP 2- step Polymer Detection System, Cell Marque). Diaminobenzadine (DAB-057, Zytomed Systems) was applied for five (CCR2, CD25), seven (iNOS) or ten (CD4, CD7, CD8, CD14, CD25, CD68, CD86, CD163) minutes and cell nuclei were counterstained using hemalum solution acid and mounted. As a positive control we used tonsil tissue.

In order to determine c-jun expression, we stained the slides with c-jun antibody (#9165S, Cell Signaling, 60A8, dilution: 1:600) and S100 (ab4066, abcam, clone: 4C4.9, dilution: 1:100). Antibodies were used diluted in antibody dilution buffer (DCS Innovative Diagnostik Systeme, Hamburg, Germany), and incubated overnight at 4°C. We visualized protein expression using the secondary antibodies AlexaFluor488 (Invitrogen, A32723, Ak-No 210) and AlexaFluor555 (Invitrogen, A32732, Ak-No 208) in a 1:1000 dilution and incubated the slides over night at 4°C. Finally, the slides were mounted using Fluoroshield mounting medium containing DAPI (Abcam, Cambridge, UK). Again, we used tonsils as positive controls.

Five representative fields of view per slide were photographed with the LEICA DMI 3000 B microscope with standardized settings at a 40× amplification and analyzed for staining intensity utilizing the batch processing function of the open-source program Fiji (ImageJ 1.53q) (20, 21). The macro underwent validation through a comparative analysis with manually counted samples consisting of five slides per staining. Additionally, validation was conducted under the supervision of our experienced neuropathologist (CMM).

### 2.3 Statistical analysis

Statistical analysis was performed using GraphPadPrism 9 software (GraphPad Software, San Diego, USA). Descriptive statistics was performed to evaluate patient data. Hemalaun/DAPI staining was counted by a coded Macro (**Supplementary Materials 1**, **2**), while DAB/c-jun expression was determined by two independent investigators. Statistical significance was defined by unpaired 2-tailed t-tests, and by ANOVA. P < 0.05 was considered to be significant. In the box plots the boxes represent the median with the 25% and 75% quartile and the whiskers the minimum and maximum of the data set. Volumetric analysis (cm³) was conducted on MRI scans both before and after surgical intervention. The tumor growth rate was determined by quantifying the disparity between two MRI scans prior to surgery. Subsequently, a linear regression analysis was executed, employing SPSS 29 (IBM SPSS Statistics, Chicago, USA), with antigen expression serving as the dependent variable. We defined slowly growing tumors as 2 mm growth per year and rapid growing tumors as > 2 mm per year.

## 3 Results

### 3.1 Patients’ characteristics

The analysis comprised forty patients, all of whom were histopathologically confirmed to have VS. Among the participants, 27 were female, and 13 were male, with an average age of 46 years ± 17 years. 20 of the patients had a concurrent diagnosis of NF2; their average age was 36 years ± 13 years (**Table 1**).

**Table 1:** Patient characteristics.

	NF2-associated VS		Sporadic VS	
Male: Female	6 : 14		8 : 12	
Age	36 ± 13		56 ± 14	
MRI progression	Slow: 10	Rapid: 10	Slow: 10	Rapid: 10
Tumor volume [cm^3^]	4.0 (0.2-25.3)	5.2 (1.1-22.5)	4.8 (0.3-15.3)	3.0 (0.2-14.7)
Tumor growth [cm^3^]	-0.5 (-9.8-2.1)	5.1 (-0.9-21.1)	0.6 (-3.0-3.0)	1.7 (-3.5-5.6)
Tumor extension	T3A: 4 T3B: 3 T4: 3	T3A: 0 T3B: 1 T4: 9	T3A: 2 T3B: 2 T4: 6	T3A: 3 T3B: 3 T4: 4
Tumor localization	5 right, 5 left	4 right, 6 left	4 right, 6 left	5 right, 5 left
Hearing	H1: 2 H2: 2 H3: 1 H4: 0 H5: 0 H6: 5	H1: 0 H2: 0 H3: 0 H4: 0 H5: 0 H6: 10	H1: 0 H2: 3 H3: 2 H4: 2 H5: 0 H6: 3	H1: 0 H2: 3 H3: 2 H4: 0 H5: 0 H6: 5
Antoni Type	A: 6* A/B: 3* B: 0*	A: 2 A/B: 8 B: 0	A: 2 A/B: 6 B: 2	A: 5 A/B: 3 B: 2
Ki67	1-2%: 6 3-5%: 4 10%: 0 15%: 0	1-2%: 5 3-5%: 3 10%: 1 15%: 1	1-2%: 4 3-5%: 6 10%: 0 15%: 0	1-2%: 0 3-5% 9 10%: 1 15%: 0

Tumor extension according to Hannover classification (19). Slow = maximum progress of 2 mm per year, rapid = more than 2 mm growth per year. Initial tumor volume and tumor growth are provided in cm^3^, Mean and minimum/maximum values are provided. Hearing function according to Hannover classification (19). Histology by Antoni type A, B, A/B, NF2 = NF2-related schwannomatosis, VS = vestibular schwannoma, * signifies missing data. Age is provided as mean, tumor volume as median.

### 3.2 Spatial distribution of cellular TME components

**Figure 2:**
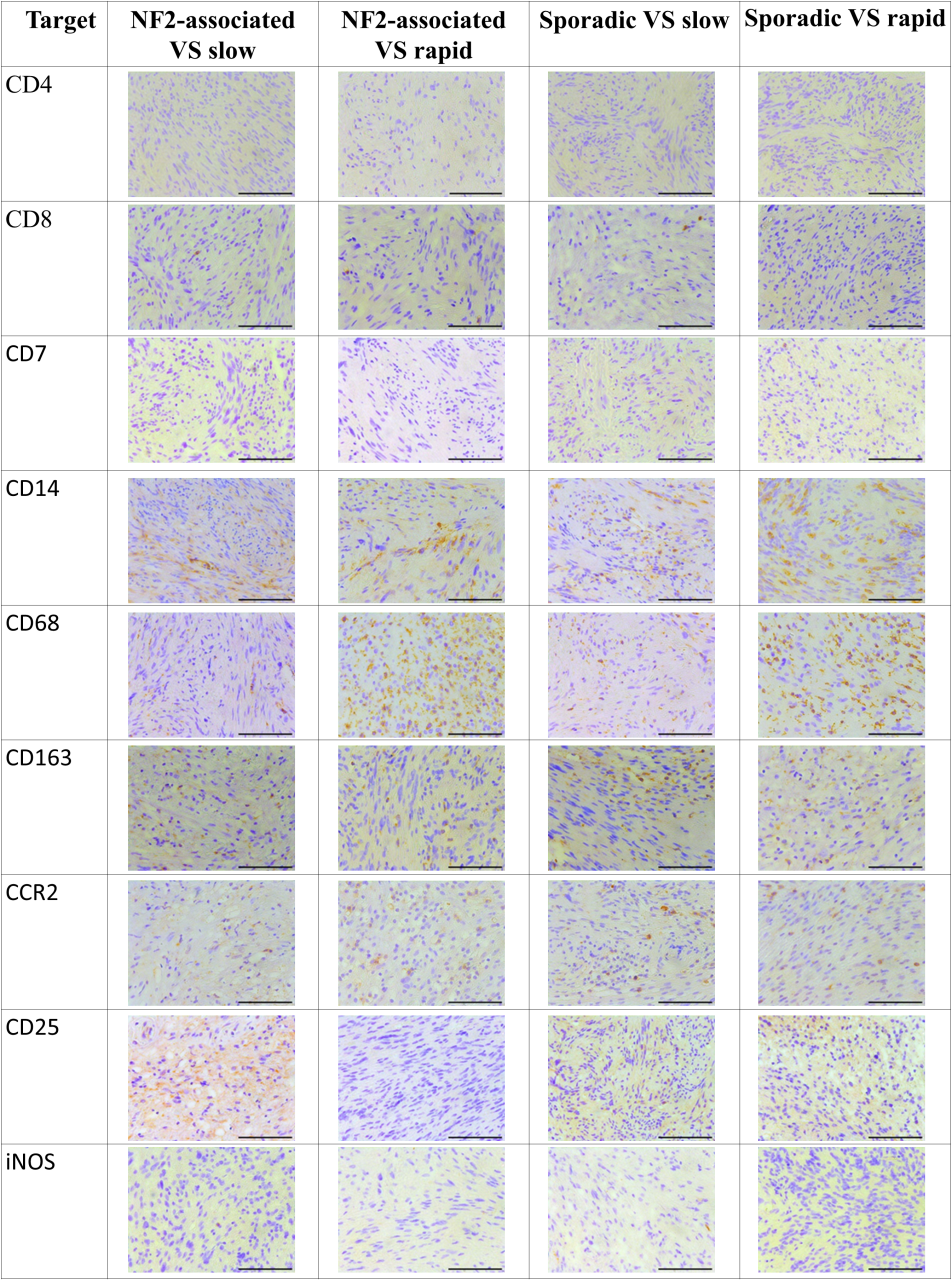
Distribution of cellular tumor environment marker expression in neurofibromatosis type 2 (NF2)-associated as well as sporadic vestibular schwannoma (VS), both distinguished by slow and rapid growth. Tissues were stained with CD4, CD7, CD8, CD14, CD25, CD68, CD163, CCR2 and iNOS antibodies. Scale bar 200 µm.

**Figure 3:**
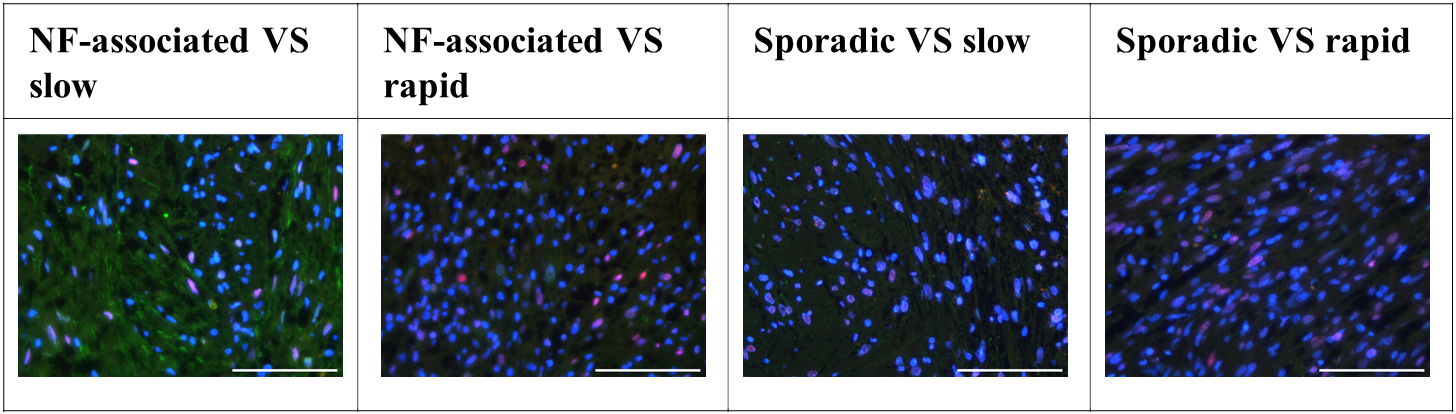
Distribution of c-jun expression in neurofibromatosis type 2 (NF2)-associated as well as sporadic vestibular schwannoma (VS), both distinguished by slow and rapid growth. Scale bar 200 µm.

The examination of tissue slides by the senior author (MB) and an experienced neuropathologist (CMM) revealed distinct patterns of immune cell marker expression and distribution. CD4- and CD7-cells were notably scarce within the samples. Conversely, CD8-positive cells were detected, but their numbers were minimal. These CD8-positive cells exhibited a solitary, homogenous distribution throughout the tissue slices, primarily concentrated within regions characterized by lower cellular density, rather than within the palisade-like areas. In contrast, CD25-positive cells were found in abundance, particularly in rapidly growing sporadic VS. CD25 distribution was uniformly homogenous, primarily observed within areas of lower cellular density. Importantly, there was no discernible accumulation of CD25-positive cells along the tissue periphery or around vascular structures. CD68-positive cells were notably abundant and uniformly distributed throughout the tissue samples, with a concentration within less densely populated areas. No evident accumulation of CD68-positive cells was observed along the tissue edges or around vascular elements. CD14 and CD163 expressing cells exhibited a high prevalence within regions of lower cellular density, as well as between palisade structures. CD163-positive cells were sporadically detected in the vicinity of vascular structures. In terms of chemokine receptors, CCR2 displayed low expression, with solitary cells exhibiting a homogenous distribution, particularly within less densely populated regions. Conversely, iNOS expression was nearly negligible, indicating minimal presence in the examined tissue samples. These findings collectively characterize the spatial distribution and abundance of various immune cell markers in the tissue slices, providing valuable insights into the immunological landscape of the TME (**Figure 2**). C-jun expression levels were notably higher in rapidly growing NF2-associated vestibular schwannoma (VS) and in patients with slowly growing sporadic VS. Furthermore, a significant disparity in c-jun expression was evident between NF2-associated and sporadic VS, as illustrated in **Figure 3**.

### 3.3 Analysis of cellular TME markers in NF-2 associated and sporadic VS

**Figure 4:**
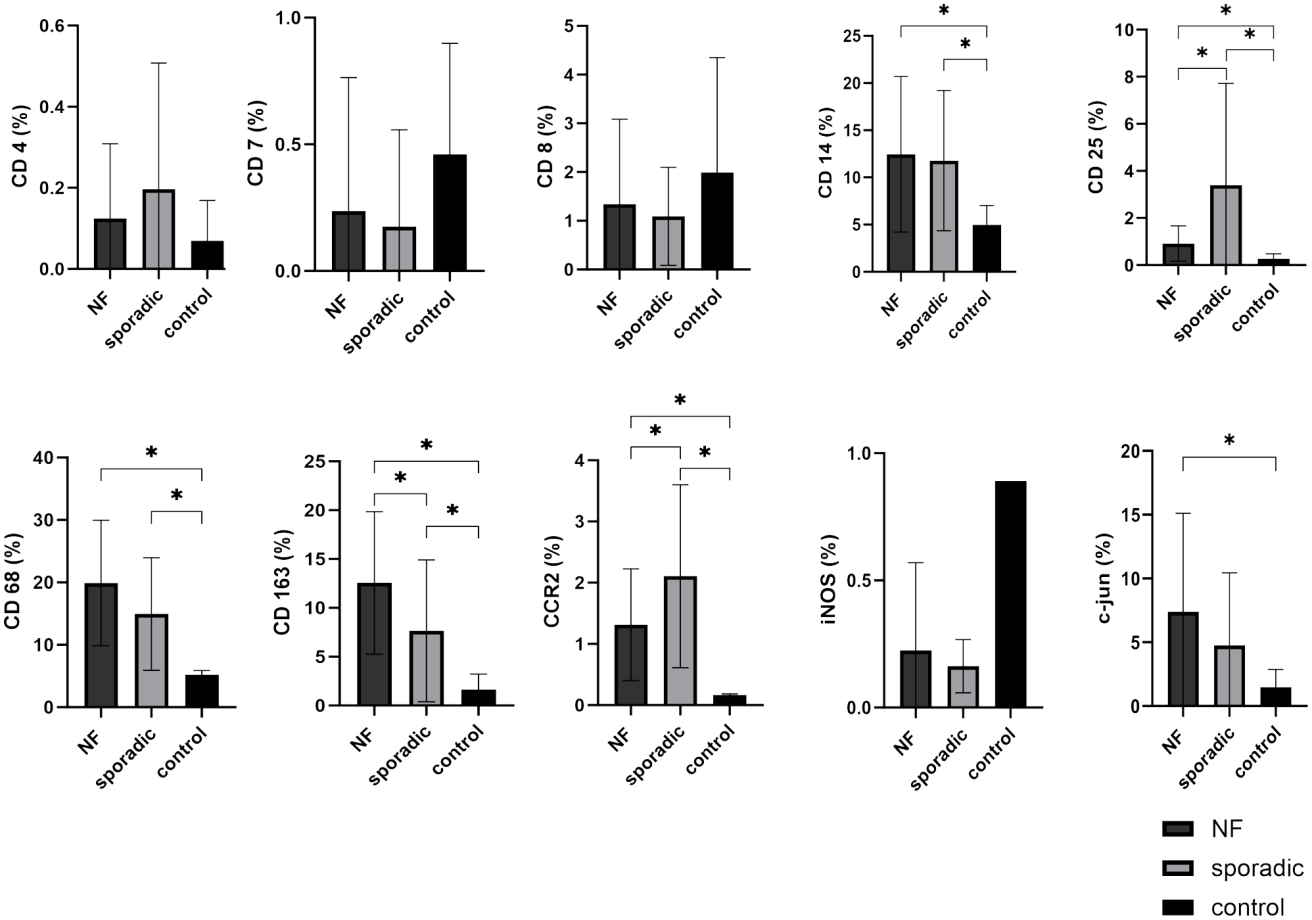
Expression of cellular tumor microenvironment markers in neurofibromatosis type 2 (NF2)-associated and sporadic vestibular schwannomas (VS). A panel of the cellular markers CD4, CD7, CD8, CD14, CD25, CD68, CD163, CCR2, iNOS, and c-jun was subjected to immunohistochemical staining. The relative marker expression levels were quantified in relation to the total cell count for both NF2-associated (NF; depicted in dark grey) and sporadic (depicted in light grey) (VS, with control nerves shown in black for reference. Statistically significant differences are indicated by asterisks. Whiskers are representing standard deviation values.

We assessed the expression levels of key immune cell markers CD4, CD8, and CD7 within VS tissue and compared them to control nerve tissue. Our findings revealed that CD4, CD8, and CD7 exhibited low expression levels in VS and did not demonstrate statistically significant increases when compared to control nerve tissue. Furthermore, there were no significant differences detected between NF2-associated and sporadic VS in terms of these immune cell markers (**Figure 4**).

Conversely, monocyte and macrophage cell markers CD14, CD68, CD163, and CCR2 were expressed at higher levels in VS compared to the T-cell markers. CD14 (p = 0.0187), CD68 (p < 0.0001), CD163 (p = 0.0006), and CCR2 (p < 0.0001) exhibited significantly elevated expression levels in VS compared to control nerve tissue. Notably, both CCR2 (p < 0.0001) and CD163 (p = 0.0397) displayed significant differences between NF2-associated and sporadic VS. CCR2 expression was higher in sporadic VS, whereas CD163 had higher expression in NF2-associated VS relative to sporadic cases. Interestingly M1 marker iNOS showed a very low expression level in both sporadic and NF2-associated VS, the expression pattern is rather similar to the lymphocyte expression than to the aforementioned macrophage/monocyte expression. Furthermore, the marker CD25, representative of regulatory T-cells, exhibited higher expression levels in sporadic VS in comparison to NF2-associated VS (p = 0.0236) and control nerve tissue (p = 0.0058).

In addition, we investigated the expression of c-jun, a marker indicative for Büngner cells - Schwann cells in repair mode. We observed high expression of c-jun in VS, particularly in NF2-associated VS when compared to control nerve tissue (p = 0.0124). The localization of c-jun was primarily observed within cell nuclei. It is noteworthy that approximately one-third of the tissue slices contained variations in the density of positively stained cells, with some areas exhibiting higher and others lower levels of positive staining.

### 3.4 Analysis of cellular TME markers in relation to VS growth dynamics

**Figure 5:**
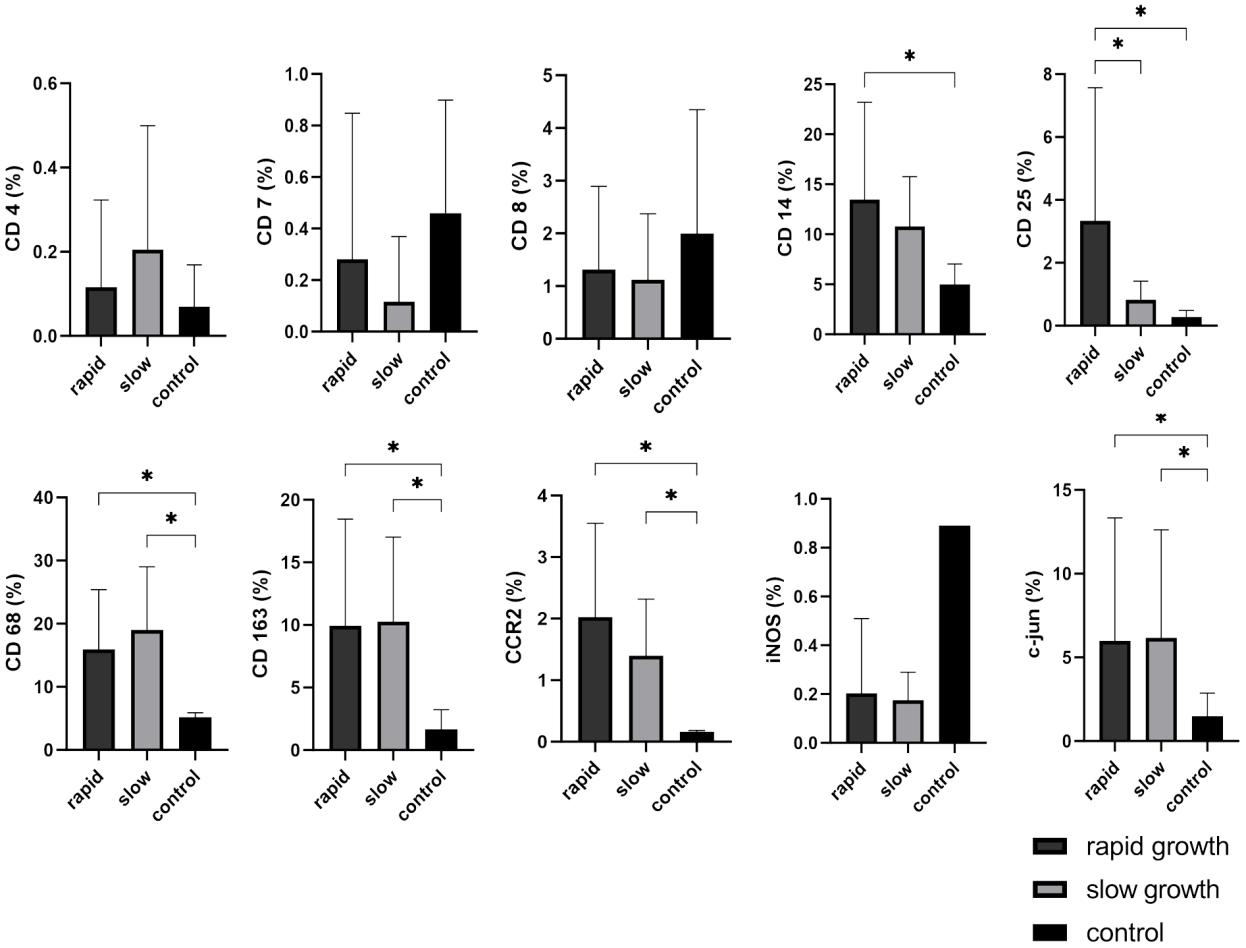
Expression of cellular tumor environment markers in relation to vestibular schwannoma (VS) growth dynamics: A panel of the cellular markers CD4, CD7, CD8, CD14, CD25, CD68, CD163, CCR2, iNOS, and c-jun was subjected to immunohistochemical staining. The relative marker expression levels were quantified in relation to the total cell count for both rapidly growing (depicted in dark grey) and slowly growing (depicted in light grey) VS, with control nerves shown in black for reference. Statistically significant differences are marked by asterisks. Whiskers are representing standard deviation values.

CD25 was highly expressed in rapidly growing VS (p = 0.016), whereas there was no difference between slowly-growing VS and control nerves. Hence, it holds promise as a potential marker for assessing tumor growth dynamics. The markers CD68, CD163, CCR2, and c-jun did not show differences between rapidly and slowly growing VS, but were significantly increased compared to healthy vestibular nerves (slow-growth: CD68 p = 0.0001, CD163 p = 0.0035, CCR2 p < 0.0001, c-jun p = 0.0278; rapid-growth: CD68 p < 0.0001, CD163 p = 0.0027, CCR2 p < 0.0001, c-jun p = 0.0393). In contrast, CD14 displayed significantly higher expression only in rapidly progressing VS compared to control nerves (p = 0.0111). Notably, iNOS demonstrated downregulation in VS compared to healthy control nerves. However, statistical significance could not be reached due to the limited number of control nerves, and no discernible impact of growth dynamics was observed (**Figure 5**).

**Figure 6:**
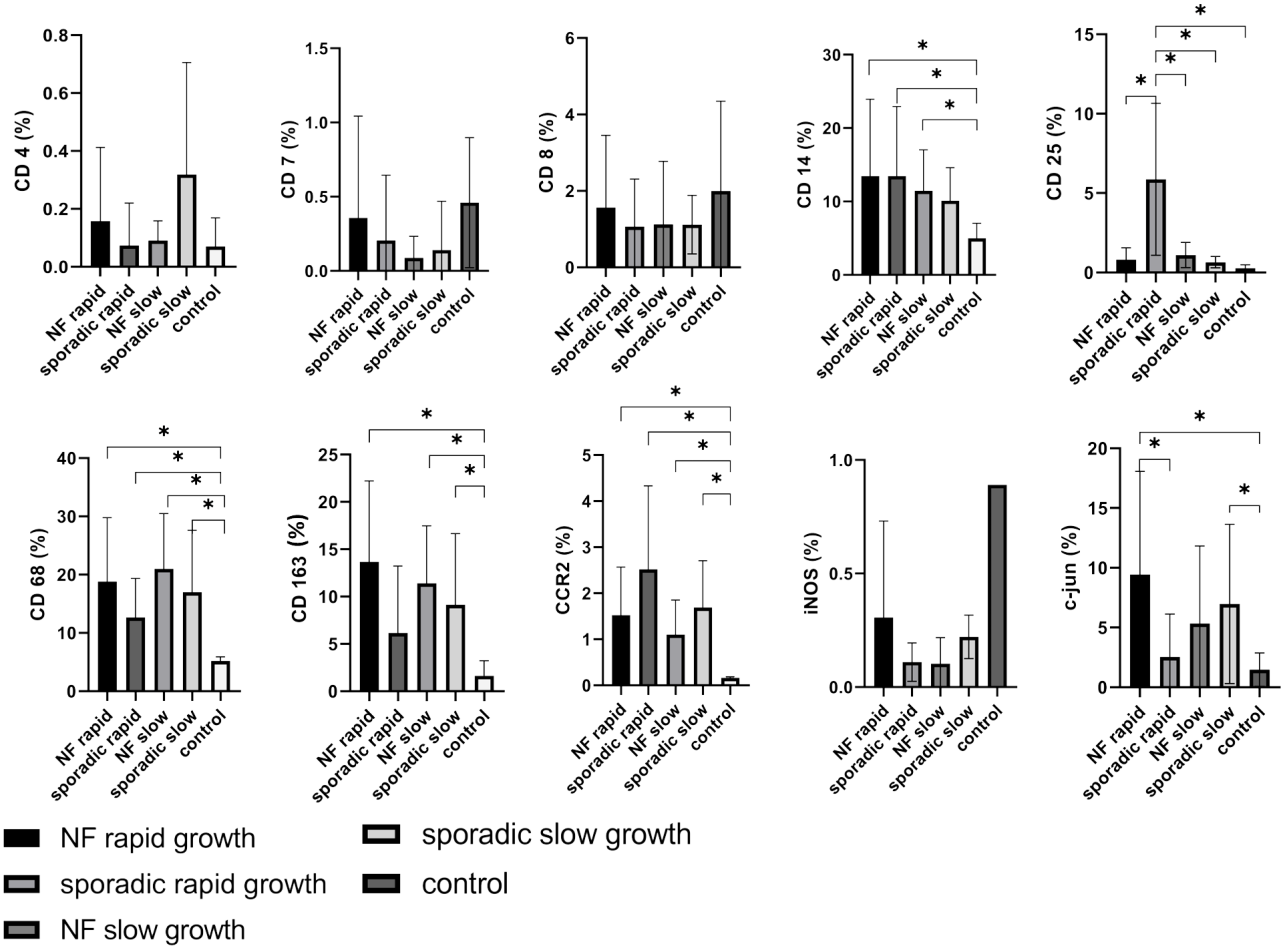
Expression of cellular tumor environment markers in relation to growth dynamics of both neurofibromatosis type 2 (NF2)-associated and sporadic vestibular schwannomas (VS): A panel of the cellular markers CD4, CD7, CD8, CD14, CD25, CD68, CD163, CCR2, iNOS, and c-jun was subjected to immunohistochemical staining. The relative marker expression levels were quantified in relation to the total cell count for rapidly (depicted in dark grey) and slowly growing (depicted in light grey) sporadic VS as well as for rapidly (depicted in black) and slowly growing (depicted in medium grey) NF2-associated VS, with control nerves shown in white for reference. Statistically significant differences are marked by asterisks. Whiskers represent standard deviation values.

For subanalysis between rapid and slow growth patterns in VS, lymphocyte markers such as CD4, CD7, and CD8 were investigated, but our findings did not reveal any statistically significant differences when compared to control nerves (**Figure 6**). In contrast, CD25 displayed an intriguing pattern. It was found to be significantly higher in patients with rapidly growing sporadic VS compared to those with NF2-associated rapid growth (p = 0.0086), NF2-associated slow growth (p = 0.00119), sporadic VS slow growth (p = 0.007), and control nerves (p = 0.0049) (**Figure 6**).

The growth rate was determined through volumetric analysis, involving two MRI scans conducted one year apart before surgery. The calculated growth rate was 1.86 cm³ with a margin of error of ±4.31 cm³ (ranging from a minimum of -9.8 cm³ to a maximum of 21.1 cm³), corresponding to a monthly growth rate of 0.2cm³ with a margin of error of ±0.46 cm³ (ranging from a minimum of -1.23 cm³ to a maximum of 1.86 cm³). In the course of conducting a linear regression analysis, wherein the growth rate was considered as an independent variable and antigen expressions were regarded as dependent variables, it was observed that the tumor growth rate did not exhibit a statistically significant impact on any of the antigens.

As previously mentioned, markers associated with monocytes and macrophages, including CD14 (NF2-associated rapid: p = 0.0424; sporadic VS rapid: p = 0.0310; NF2-associated slow: p = 0.0368), CD68 (NF2-associated rapid: p = 0.0034; sporadic VS rapid: p = 0.0102; NF2-associated slow: p = 0.0005; sporadic VS slow: p = 0.0067), CD163 (NF2-associated rapid: p = 0.0022; NF2-associated slow: p = 0.0023; sporadic VS slow: p = 0.0186), and CCR2 (NF2-associated rapid: p = 0.0026; sporadic VS rapid: p = 0.0027; NF2-associated slow: p = 0.0034; sporadic VS slow: p = 0.0011), exhibited significantly elevated when compared to control samples. It is noteworthy that the M1 marker iNOS exhibited a notably low expression level in both sporadic and NF2-associated vestibular schwannoma (VS), regardless of the tumor growth rate. This expression pattern bears a closer resemblance to lymphocyte expression patterns than to the previously mentioned macrophage/monocyte markers. C-jun demonstrated increased expression in rapidly growing NF2-associated VS (p = 0.0205), as well as in patients with slowly growing sporadic VS (p = 0.0412) compared to the control nerve, and also a significant difference between NF2-associated and sporadic VS (p = 0.037) (**Figure 6**).

### 3.5 Analysis of cellular TME markers in relation to hearing function

**Figure 7:**
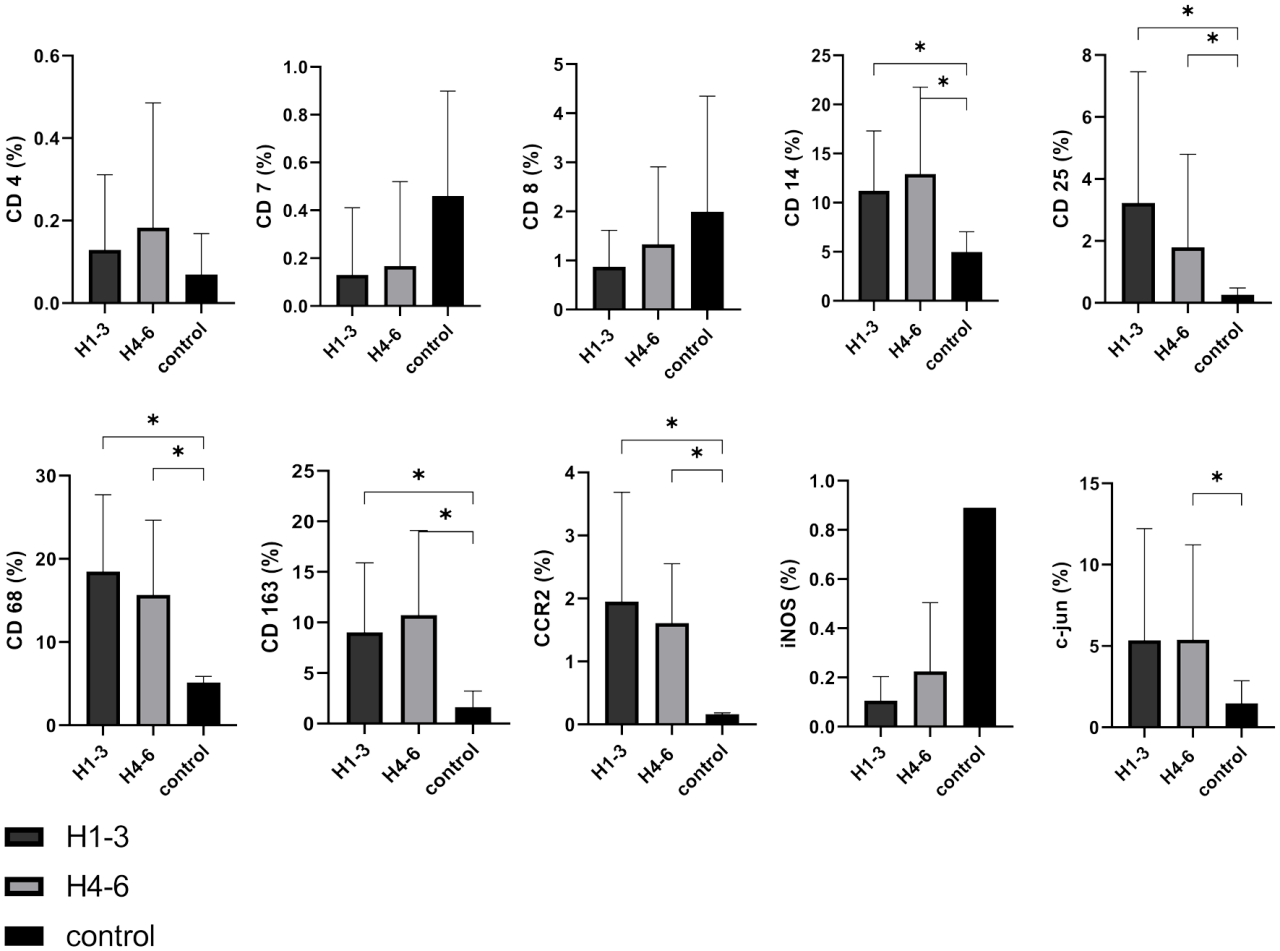
Expression of cellular tumor environment markers in relation to hearing function: A panel of the cellular markers CD4, CD7, CD8, CD14, CD25, CD68, CD163, CCR2, iNOS, and c-jun was subjected to immunohistochemical staining. H1-3 according Hannover classification = good hearing with a maximum hearing loss of 60 dB, H4-6 according Hannover classification = bad hearing with more than 60 dB hearing loss up to deafness. The relative marker expression levels were quantified in relation to the total cell count for both good hearing function (depicted in dark grey) and bad hearing function (depicted in light grey) in vestibular schwannoma patients and control nerves shown in black for reference. Statistically significant differences are indicated by asterisks. Whiskers represent standard deviation values.

We observed that CD4, CD7, and CD8 were expressed at low levels in VS, and consequently, no correlation with hearing function was discernible. It is of significance that the M1 marker iNOS displayed a lower expression level, irrespective of hearing function. This expression profile more closely aligns with the patterns observed in lymphocytes rather than with the macrophage/monocyte markers. Conversely, the markers CD14 (H1-3 p = 0.0394; H4-6 p = 0.041), CD 25 (H1-3 p = 0.0342; H4-6 p = 0.0341), CD68 (H1-3 p < 0.0001; H4-6 p < 0.0001), CD163 (H1-3 p = 0.0072; H4-6 p = 0.0016), and CCR2 (H1-3 p = 0.0041; H4-6 p < 0.0001) exhibited significantly higher expression in comparison to control tissue across both hearing subgroups. C-jun was significantly higher expressed in patients with bad hearing function compared to controls (p = 0.0479). However, noteworthy is the absence of significant differences between patients with good (H1-3) and poor (H4-6) hearing function (**Figure 7**). This indicates that none of these markers has a causal link to hearing impairment.

## 4 Discussion

The present analysis offers a comprehensive examination of the TME in sporadic and NF2-associated VS, encompassing some clinical parameters and a wide array of immune cell markers. Emerging evidence underscores the increasing importance of immune cells within the microenvironment. Contrary to the conventional notion that only mutated Schwann cells bear relevance in VS pathogenesis, our findings reveal a substantial presence of immune cells, particularly macrophages, in these benign tumors (22).

Macrophages (CD14+, CD68+) are notably abundant in VS, particularly in growing tumors, larger lesions, those displaying a higher proliferation index, and cases characterized by prolonged symptom duration (23). M2 macrophages (CD163+) have a pivotal mechanistic role in tumor growth, angiogenesis, and tumor invasion, primarily through their capacity to degrade the extracellular matrix (8, 24–26). In our cohort, we found a significant higher expression of macrophage markers in both NF2-related and sporadic VS, which is mirrored by an analysis of bulk transcriptomic data of three microarray data sets comparing the gene expression profiles of sporadic and NF2-related VS (27). In this investigation, no significant differences in signaling pathways, gene expression, cell type abundance or imaging mass cytometry staining between NF2-related and sporadic VS could be found. Intriguingly, our cohort identified a substantial TAM marker presence not only in NF2 patients but also in sporadic cases when compared to healthy vestibular nerves. Expression of macrophage markers was lower (although not significantly) in sporadic compared to NF2-related VS, indicating potentially different macrophage infiltration between these tumor types in line with the findings provided by Lewis et al. (28). This study aimed to conduct a comparative analysis of the TME of sporadic and NF2-related VS using a comprehensive approach involving imaging and tissue analysis. Further examination revealed a notable increase of proliferation marker Ki67 regions characterized by high TAM density compared to those with low-density regions highlighting a potential link between macrophage infiltration and cellular proliferation. Several other Studies have linked higher TAM density with increased microvessel density and elevated levels of macrophage colony stimulating factor (M-CSF), suggesting potential therapeutic targets (29). Additionally, there’s evidence of spatial correlation between TAM density and expression of vascular endothelial growth (VEGF) and its receptor VEGFR-1, indicating a role for VEGF pathway in TAM recruitment. Investigation into sporadic VS growth has shown a positive correlation between immune cell factors such as CD45 and CD68 expression and tumor size and growth rate, as well as elevated CD163 expression in rapidly growing tumors (29). These findings underscore the significance of TAMs in VS progression and suggest potential therapeutic strategies targeting TAMs and angiogenesis pathways. However, our analysis could not replicate a correlation with tumor growth dynamics, constrained by the limitations of sample size and the retrospective nature of the study. None of the immune markers demonstrated a correlation with hearing function as an indicator of tumor induced neuropathy, a finding consistent with the majority of published literature, which highlights only isolated targets such as NLRP3 inflammasomes as correlating with hearing function (23). Regulatory T-cells, though infrequently investigated in the context of VS (30) exhibit a correlation with tumor growth. Our analysis identified CD25, a marker for regulatory T-cells, as highly expressed in rapidly growing sporadic VS in our cohort.

The significance of immune cells as novel targets for systemic interventions is gaining prominence. Potential avenues include the use of aspirin as a COX2 inhibitor, re-education of M2 macrophages to M1 macrophages, antagonists of IL-1 and TNFα, checkpoint inhibitors, PD-L1 inhibitors, or Bevacizumab as an angiogenesis inhibitor. Programmed death-1 (PD-1) is a surface protein found on cytotoxic T-cells, while its counterpart, Programmed death-ligand 1 (PD-L1), is frequently present on the surface of tumor cells in various malignancies. Binding of PD-L1 to PD-1 inhibits the immune response mediated by cytotoxic T-cells (30). Although PD-L1 expression was not evaluated in VS in our study, however, cytotoxic T-cells were present in both sporadic and NF2-associated VS. Targeting this interaction could be a promising avenue for future therapeutic interventions in VS. Regulatory T cells, identified by the expression of CD4, CD25, and Foxp3 proteins, play a pivotal role in tumor progression by suppressing immune responses specifically targeting tumors. In patients with NF2, the abundance of Foxp3-positive regulatory T cells in progressing schwannomas significantly exceeded that in non-progressing cases, indicating a potential link between the presence of these cells and tumor growth (30). While our analysis did not reveal a significant difference in CD25 expression between rapidly and slowly growing VS, there is a noticeable trend towards increased CD25 expression in rapidly growing sporadic VS. This suggests the possibility of targeting CD25-positive regulatory T cells as a potential treatment strategy for rapidly growing sporadic VS. Furthermore, immune cells could serve as prognostic biomarkers if imaging methods capable of *in vivo* quantification, such as PET-CT for Iba 1 (an M2 marker), can be developed. Such biomarkers could aid in assessing disease progression, especially in cases of partial resection (23). Notably, Schwann cells in repair mode, as indicated by c-jun expression, may also play a pertinent role in pathogenesis. Schulz and colleagues observed a modest increase in c-jun expression, indicative of Schwann cell immaturity, in crushed nerves within an orthotopic mouse model featuring both homozygous and heterozygous nf-2 knock-out mice, as compared to wild-type animals (31). In our investigation, we witnessed a significant overexpression in rapidly growing sporadic VS, indicating repair cells in rapidly growing tumors. Interestingly, these findings are mirrored by the expression of regulatory T-cells. The therapeutic implications and functional roles of these Schwann cells remain an enigma, awaiting further investigation.

Our study represents a comprehensive investigation into the cellular expression of TME within VS, encompassing both NF2-associated and sporadic cases. By analyzing a substantial cohort of 40 patients, we aimed to provide a thorough comparative analysis of the TME in these distinct clinical contexts. It is important to note that while our study contributes valuable insights to basic research, it does not extrapolate findings directly applicable to clinical diagnostic, prognostic, or therapeutic decisions. Although our primary focus was not on clinical applications, we acknowledge the potential therapeutic implications of targeting the TME for patients. However, our study did not specifically investigate therapeutic interventions. Our research involved an in-depth examination of various clinical parameters by assessing immune components of the TME through immunohistochemistry. This included the evaluation of T-cell markers (CD4, CD8), NK cells (CD7), regulatory T-cells (CD25), and monocytes/macrophages (CD14, CD68, CD163), alongside innovative markers such as iNOS (as a macrophage marker) and c-jun (as a Büngner cell marker). Through meticulous analysis, we were able to establish significant correlations between immune cell populations and various clinical parameters. However, it is important to acknowledge the limitations of our study. Analysis was performed using paraffin-embedded tissue, which may introduce changes compared to fresh tissue samples. Additionally, immunohistochemistry is a semi-quantitative method, and despite our efforts to ensure methodological rigor, inherent limitations and potential for error exist. While alternative methods such as flow cytometry could have been considered, they would have resulted in the loss of spatial distribution information for the markers within the tissue. In summary, our study contributes to the understanding of the TME in VS, emphasizing the complexity of immune cell interactions and their potential implications for clinical outcomes. However, further research incorporating complementary methodologies and larger patient cohorts is warranted to elucidate the clinical relevance of our findings.

In conclusion, immune cells play a pivotal role in the pathogenesis of VS, both in the context of NF2-associated and sporadic cases, with a particular emphasis on monocytes and macrophages. Notably, our analysis revealed significant differences in the expression of CCR2 and CD163 between NF2-associated and sporadic VS. Downregulation of iNOS, a marker associated with M1 macrophages, was observed in VS, although statistical significance was not reached. Furthermore, CD25, a marker indicative for regulatory T-cells, exhibited significant differences between slowly and rapidly progressing VS, suggesting its potential utility as a biomarker for assessing tumor growth dynamics. These findings underscore the emerging significance of immune cells as promising candidates for both biomarker development and systemic therapeutic approaches in the context of VS. Further prospective investigations are warranted to elucidate the functional implications of these immune cell markers and their potential as targets for therapeutic intervention.

## Data availability statement

The raw data supporting the conclusions of this article will be made available by the authors, without undue reservation.

## Ethics statement

The studies involving humans were approved by Ethikkommission der Universität Würzburg. The studies were conducted in accordance with the local legislation and institutional requirements. The participants provided their written informed consent to participate in this study.

## Author contributions

VN: Data curation, Formal analysis, Funding acquisition, Investigation, Methodology, Project administration, Resources, Supervision, Visualization, Writing – original draft, Writing – review & editing. DZ: Formal analysis, Investigation, Validation, Writing – review & editing. CR: Investigation, Visualization, Writing – original draft. DK: Conceptualization, Methodology, Writing – review & editing. RN: Formal analysis, Validation, Writing – review & editing. ER: Investigation, Methodology, Writing – review & editing. CMM: Conceptualization, Methodology, Resources, Supervision, Validation, Writing – review & editing. R-IE: Conceptualization, Methodology, Resources, Supervision, Writing – review & editing. CM: Conceptualization, Funding acquisition, Methodology, Supervision, Writing – review & editing. ML: Conceptualization, Methodology, Supervision, Writing – review & editing. CH: Conceptualization, Methodology, Resources, Supervision, Writing – original draft. MB: Writing – original draft, Formal analysis, Funding acquisition, Investigation, Resources, Supervision, Visualization, Writing – review & editing.
