# Supplementary material for: Analysis of tumor microenvironment composition in vestibular schwannomas: insights into NF2-associated and sporadic variations and their clinical correlations
Source: Front Oncol. 2024 May 16;14:1340184. doi: 10.3389/fonc.2024.1340184 (PMC11137168; doi:10.3389/fonc.2024.1340184)
Supplement: Supplementary file 2 [file DataSheet_2.docx]

**Supplement 2: Macro for hemalaun evaluation in immunohistochemical staining**

input = getDirectory("Input directory");

output = getDirectory("Output directory");

Dialog.create("File type");

Dialog.addString("File suffix: ", ".tif", 5);

Dialog.show();

suffix = Dialog.getString();

processFolder(input);

function processFolder(input) {

list = getFileList(input);

for (i = 0; i < list.length; i++) {

if(File.isDirectory(input + list[i]))

processFolder("" + input + list[i]);

if(endsWith(list[i], suffix))

processFile(input, output, list[i]);

}

}

function processFile(input, output, file) {

open(input + File.separator + file);

name=getTitle();

run("8-bit");

//run("Brightness/Contrast...");

setMinAndMax(0, 255);

run("Apply LUT");

run("Gaussian Blur...", "sigma=4");

setAutoThreshold("Otsu dark");

setOption("BlackBackground", true);

run("Convert to Mask");

run("Watershed");

run("Analyze Particles...", "size=0.03-1.00 circularity=0.65-1 show=Outlines exclude summarize");

run("Close All");

}
